# NRF2 and p53: Januses in cancer?

**DOI:** 10.18632/oncotarget.754

**Published:** 2012-11-19

**Authors:** Barak Rotblat, Gerry Melino, Richard A. Knight

**Affiliations:** ^1^ Medical research Council, Toxicology Unit, Leicester University, Leicester, UK; ^2^ Biochemistry IDI-IRCCS Laboratory, Department of Experimental Medicine and Surgery, University of Rom ‘Tor Vergata’, Rome, Italy

**Keywords:** P53, p73, TIGAR, p21, NQO1, NADPH, ROS, metabolism, proteasome, KEAP1, MDM2, chemotherapy, BSO, piperlongumine, BPTES, brusatol

## Abstract

The transcription factor nuclear factor (erythroid-derived 2)-like 2, also known as NFE2L2 or NRF2, is a master regulator of the anti-oxidative stress response and positively controls the expression of a battery of anti-oxidative stress response proteins and enzymes implicated in detoxification and glutathione generation. Although its detoxifying activity is important in cancer prevention, it has recently been shown that cancer cells also exploit its protective functions to thrive and resist chemotherapy. NRF2 was also shown to the pentose phosphate pathway and glutaminolysis, which promotes purine synthesis for supporting rapid proliferation and glutathione for providing anti-oxidative stress protection. Evidence obtained from cancer patients and cell lines suggest that NRF2 is highly active in a variety of human cancers and is associated with aggressiveness. p53 is a tumor suppressor that also promotes an anti-oxidative stress metabolic program and glutaminolysis. Here we will discuss the similarities between NRF2 and p53 and review evidence that p53 might be exploited by cancer cells to gain protection against oxidative stress, as is the case for NRF2. We discuss findings of co-regulation between these transcription factors and propose possible therapeutic strategies that can be used for treatment of cancers that harbor WT p53 and express high levels of NRF2.

## INTRODUCTION

Reactive oxygen species (ROS) are an integral part of life and are a byproduct of respiration and exposure to the environment [1, 2]. When in excess, ROS can damage DNA, proteins and lipids and promote mutations that may contribute to onset of a wide spectrum of human diseases [3-5] particularly diseases that are associated with aging [6-8] such as neurodegeneration [9-12] and cancer [13-15]. To maintain redox and prevent aberrant ROS accumulation molecular-genetic mechanisms have evolved that sense and respond to oxidative stress. These genetic programs involve expression of proteins that directly detoxify ROS [16], or that are part of metabolic programs that generate anti-oxidants such as glutathione [16-24].

While these mechanisms are important to prevent disease onset, it is now becoming evident that the same mechanisms are being exploited by cancer cells in order to survive and thrive under oxidative stress insults and to armor themselves against therapeutic interventions that rely on oxidative stress as their mechanism of action [25-29]. Here we will review two transcription factors that promote transcriptional-metabolic anti-oxidative stress programs, NRF2 and p53. Both nrf2 and p53 knockout mice show enhanced susceptibility to induced or spontaneous tumors, and are therefore tumor suppressors, but have now been implicated as cancer promoting in some pathological contexts. We will discuss evidence for crosstalk between these transcription factors and possible therapeutic strategies arising from these observations.

### The NRF2 anti-oxidative stress transcriptional program plays an important role in tumor prevention

The NRF2-KEAP1 molecular system is a sensor of oxidative stress [30]. Under ambient conditions, NRF2 interacts with KEAP1, an adaptor molecules which directs its targets to the CUL3 E3 ligase [31] for ubiquitylation and subsequent degradation by the proteasome [32, 33]. This mechanism results in low basal NRF2 levels and activity. Under oxidative stress, specific KEAP1 cysteines become oxidized resulting in disruption of the KEAP1-NRF2 complex, leading to a release of NRF2 from KEAP1 inhibition and promoting its stabilization [34, 35]. Newly synthesized NRF2 then translocate to the nucleus [36, 37] to transcribe genes encoding a battery of anti-oxidant proteins [38] such as Heme Oxygenase (*HMOX1*) [39], NAD(P)H dehydrogenase (quinine; *NQO1*) [40] and key enzymes in the glutathione biosynthesis/recycling pathway such as γ-glutamylcysteine ligase (GCL) [7, 41, 42], collectively known as phase II detoxifying enzymes [43].

ROS can promote tumor initiation by damaging DNA that will lead to mutations and by augmenting signaling pathways that promote cell growth and proliferation [44]. It has long been observed that anti-oxidants and some natural compounds can be beneficial in preventing cancer initiation [45]. It was hypothesized that the protective activity of these compounds may be linked to their ability to increase expression of endogenous anti-oxidants, more specifically, the phase II detoxifying enzymes [46]. This principal was demonstrated using genetic approach where the chemo-protectant oltipraz induced phase II detoxifying enzymes and reduced cancer incidents in *Nrf2* WT but not KO mice treated with a chemical carcinogen [47]. These experiments also established NRF2 as an important factor in promoting the activity of cancer preventing compounds. Similarly, other natural compounds and genetic models were shown to rely on NRF2 for their protective activity leading to the notion that activating NRF2 is an attractive strategy to prevent cancer and reduce oxidative damage [48-56].

### The p53 anti-oxidative stress transcriptional program plays a role in preventing ROS induced DNA damage and cancer initiation

**Figure 1:**
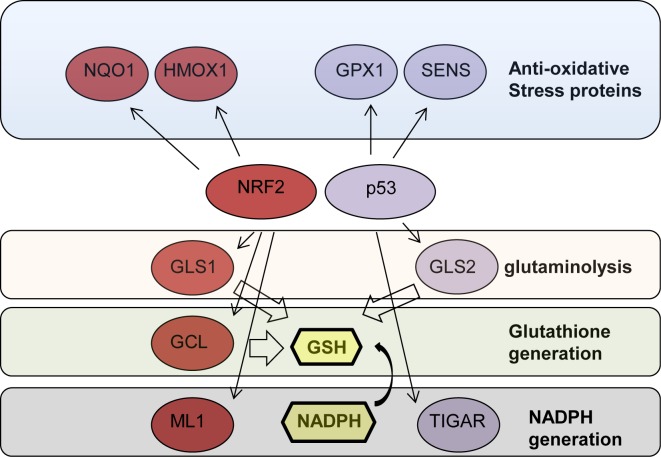
NRF2 and p53 regulate the expression of proteins involved in protection ageist oxidative stress NRF2 and p53 target genes (red and blue) that are contributing to protection against oxidative stress directly or by promoting glutathione synthesis by facilitating glutaminolysis, through direct synthesis or by facilitating NADPH production. NRF2, nuclear factor (erythroid-derived 2)-like 2; HMOX1, Heme Oxygenase 1; GCL, γ-glutamylcysteine ligase; GPX, glutathione peroxidase; NQO1, NAD(P)H quinine dehydrogenase; SENS, sestrins; GLS 1/2, glutaminase 1/2; GLH, reduced glutathione; NAPDH, Nicotinamide adenine dinucleotide phosphate.

p53 is both a positive and negative regulator of ROS [57]. p53 protein levels in the cells are tightly regulated [58-60]. In resting conditions, p53 protein is maintained at low levels by MDM2 mediated proteasomal degradation [61-66] and at this low level of expression reduces ROS levels by inducing the expression of anti-oxidative stress proteins such as SESN1, SESN2 and GPX1 [67-70]. Using a p53 KO model, it was then demonstrated that lack of expression of these anti-oxidative stress proteins is associated with increased cellular ROS which leads to increases in DNA oxidation and in the mutation rate thus promoting tumorigenesis in p53 KO mice. Later, it was also shown that p53 regulates GLS2 expression promoting glutathione generation by increasing glutaminolysis [71, 72], a metabolic process that promotes the conversion of glutamine to glutamate that is often active in cancer cells [73-77]. These findings raise the possibility that the tumor suppressor activity of p53 is related to its role in maintaining cellular redox by regulating cellular metabolism [78, 79] (Fig. 1).

### An important role for p53-mediated metabolic regulation to its tumor suppressor activity

p53 coordinates a large number of integrated transcriptional programs that result in divers biological outputs [80-84]. Deciphering the contribution of a specific aspect of p53 function to its tumor suppressor activity is one of the important questions in the p53 field [85-87]. p53 is modified by a large number of different posttranslational modifications that play an important role in the regulation of the specific cellular program that will be activated by p53 [88-90]. Manipulation of these modifications is an attractive strategy when attempting to dissect the roles of specific p53 transcriptional programs in its biological functions and pharmacological reactivation of p53 activity in cancer is an active research field [91-98]. Among other modifications, p53 is acetylated on three lysines [99-101]. Giu and coworkers studied the role of lysine acetylation to the execution of discreet p53 cellular functions related to its tumor suppressor phenotype namely, apoptosis, cell cycle arrest, senescence and the anti-oxidative stress metabolic program [102-107]. Using *p53* KO mice and cells that re-express p53 mutants, in which all three acetylated lysines were replaced by arginine (p53-3KR), they showed that, like the *p53* KO mice, mice and cells expressing p53-3KR were defective in the execution of apoptosis, cell cycle arrest and senescence. Surprisingly, p53-3KR mice did not succumb to cancer, as did the *p53 KO* mice indicating that p53-3KR retained it tumor suppressor activity. Further examination of the p53-3KR mutants revealed that they did retain the WT function in executing a transcriptional metabolic program that resulted in reducing glucose uptake, reducing glycolysis and reducing ROS generation that were associated with induction of the p53 anti-oxidative stress targets GLS2 [72] and TIGAR [108]. These findings underscore the importance of ROS regulation in the tumor suppressor activity of p53.

### Cancer cells and oncogenes hijack NRF2 for anti-oxidative stress protection

Despite the established role of NRF2 in cancer prevention [55] recent genetic evidence obtained from human cancers points to possible pro-cancer activities of NRF2. In particular it was found that there are several cancer related genetic events that prevent the degradation of NRF2 through the KEAP1-CUL3 pathway that leads to elevated NRF2 activity [33, 109]. These include somatic mutations in NRF2 that disrupt its interaction with KEAP1 [110, 111] and somatic mutations in KEAP1 that disrupt its interaction with NRF2 [112-115]. It was also found that high NRF2 expression or nuclear localization and low KEAP1 expression were associated with poor prognosis [116]. These findings, and cell based experiments, point to a model that argues that NRF2 can be exploited by cancer cells in order to curb oxidative stress and perhaps to enhance their chemo-resistance [109, 111, 116-122]

Aberrant proliferation is one of the hallmarks of cancer and oncogenes will often activate growth-promoting pathways. In a provocative paper DeNocola and collaborators showed that oncogenic K-Ras(G12D), when expressed at physiological levels, reduces ROS by increasing NRF2 that in turn promotes the expression of the anti-oxidative stress response [123]. The increase in NRF2 was promoted by the activation of the RAF pathway leading to an increase in Jun activation that enhanced *NRF2* gene expression. The authors demonstrated that NRF2 was important for K-Ras(G12V) tumorigenic functions using a murine model of mutant K-Ras driven lung and pancreatic cancer. *Nrf2* KO mice showed reduced tumor occurrence, reduced proliferation in the tumors arising in these mice and increased overall survival [123]. These findings led to the concept of a new pro-cancer activity of NRF2 in which it supports oncogene-mediated oncogenesis in addition to its proposed chemoprotective role. In support of this premise, detailed global analysis of NRF2 target genes, under both resting and NRF2 induced conditions, revealed that a substantial proportion of NRF2 targets are cell cycle promoting genes [124].

In order to support rapid proliferation, tumor cells rely on catabolic processes for generation of building blocks such as lipids, proteins and nucleic acids [125]. In a recent study, Mitsuishi et al, asked whether the increased levels of NRF2 observed in some cancer cells play a role in their rapid proliferation [126]. Using NRF2 knock down in A549 lung cancer cells, that harbor a *KEAP1* mutation and therefore express high levels of NRF2 [114], they found that NRF2 was indeed important for proliferation of these cells. In order to identify the mechanism by which NRF2 supports proliferation they used microarray analysis to identify NRF2 target genes. In addition to well-established NRF2 targets, several new target genes involved in the pentose phosphate pathway were identified suggesting that NRF2 promotes a distinct proliferation enhancing metabolic program. Indeed, high levels of NRF2 promoted the expression of proteins that support glucose flux through pentose phosphate pathway to generate purines, the building blocks of DNA and RNA, at the expense of the glycolytic pathway. Furthermore, metabolic analysis reveled that loss of NRF2 resulted in increase in cellular levels of glutamine and glutamate suggesting that NRF2 was important for catabolizing these amino acids. The authors then used a tracer study to show that NRF2 promotes glutaminolysis, the conversion of glutamine to glutamate, and directs glutamate into two metabolic pathways. One pathway is governed by up regulation of GCL that utilizes glutamate to generate glutathione. This finding is in accord with previous reports that NRF2 is important for glutathione generation [7, 41, 42]. The second pathway is induced by up regulation of ME1, where glutamate is utilized for the generation of another anti-oxidant-reducing agent, NADPH. Therefore, NRF2 induces a metabolic program that supplies building blocks to support proliferation and anti-oxidants that can protect these cancer cells from oxidative stress (Figure 1). This particular NRF2-driven pro-proliferation metabolic program was shown to be dependent on the hyperactivation of the PI3K-AKT pathway, a pathway that is typically deregulated in cancer [127, 128] providing further support to the premise that cancer cells and their oncogenic pathways utilize NRF2 to their own advantage.

### p53 in the service of cancer

The tumor suppressor activity of p53 is well documented [82, 129-132]. This activity is achieved by the employment of different pathways, spanning from gene and microRNA regulation to protein-protein interaction [130, 133-137]. However, genetic evidence obtained by analyzing patient data indicates, that in specific breast cancer sub types, WT p53 status predicts poor response to aggressive therapy [138]. One possible explanation for this observation is that WT p53 will promote cell cycle arrest and help tumor cells resists chemotherapy which targets dividing cells [139, 140]. Indeed, it was recently shown that tumors bearing WT p53 resist chemotherapy by inducing a senescence program that leads to cell cycle arrest and production of cytokines that in turn encourage the growth of drug resistant cells within the tumors [141]. These findings together with the fact that p53 promotes an anti-oxidative stress metabolic program, raise the question whether tumor cells might also use p53 in order to battle oxidative stress to gain chemo resistance, as is the case for NRF2. Indeed, p53 was shown to protect A549 cells used in the NRF2 study discussed above [126], from toxicity and radio sensitization using the metabolic inhibitor 2-Deoxy-glucose (2DG), by increasing anti-oxidative stress response and promoting oxidative phosphorylation [142, 143]. These observations, and the recent appreciation that p53 promotes a metabolic program that results in increased levels of cellular anti-oxidants [78], raise the concern that in specific pathological contexts, p53 may be exploited by cancer cells in order increase anti-oxidants to gain chemo resistance, much like NRF2.

### Cross talk between p53 and NRF2

**Figure 2:**
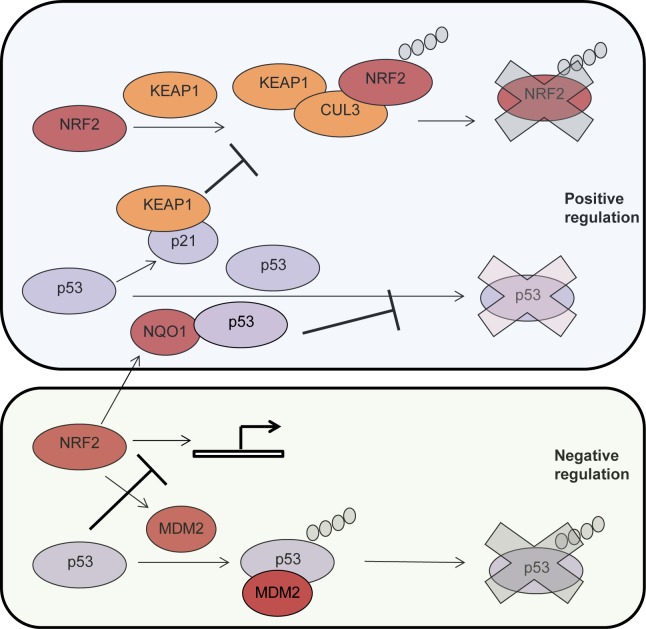
Positive and negative (up or down) co-regulation between p53 and NRF2 Top to bottom. KEAP1 interacts with NRF2 and forms a complex with the E3 ligase CUL3 that results in NRF2 ubiquitylation and degradation by the proteasome (depicted as a blue X). The p53 target gene, p21, interacts with KEAP1 and inhibits NRF2 ubiquitylation and degradation. p53 is degraded by the proteasome in a ubiquitin n dependent manner. The NFR2 target, NQO1, interacts with p53 and protects it from degradation. NRF2 target gene, MDM2, promotes p53 ubiquitylation and degradation by the proteasome. P53 is a transcriptional repressor of NRF2. NRF2, nuclear factor (erythroid-derived 2)-like 2NQO1, NAD(P)H quinine dehydrogenase; mdm2, mouse double minute 2.

It is becoming more evident that, at the functional level, p53 and NRF2 play similar roles and are both providing cells with enhanced capacity to mitigate oxidative stress. Interestingly, recent findings from the Zhang lab indicate that p21, a p53 target gene [144, 145], stabilizes NRF2 by binding to KEAP1 and interfering with its ability to promote NRF2 ubiquitylaton and proteasomal degradation [146]. On the other hand, previous findings from the Shaul lab indicate that NQO1, an NRF2 target, interacts with p53 [147] and blocks its degradation by the 20S proteasome [148], a degradation process that is independent of MDM2 and ubiquitin [59, 149] (Fig. 2). These findings support the premise of an interesting cross talk between these two transcription factors and raise the question of whether there is a positive feedback loop between NRF2 and p53 and whether cancer cells enhance their resistance to oxidative stress by utilizing this putative positive feedback loop (Fig. 2). Indeed, p53 deficient HCT116 colon carcinoma cells exhibited reduced induction of NRF2 target genes as compared with p53 proficient HCT116 cells following challenge with oxidative stress [150] suggesting that p53 may be important for NRF2 activation in cancer cells. However, this model may not be complete, as it was recently shown that *MDM2* is a transcriptional target of NRF2 through which NRF2 negatively regulates p53 [151] (Fig.2). In another study it was also shown that p53 binds to promoter elements activated by NRF2 and is therefore a transcriptional repressor of NRF2 target genes [152] (Fig. 2). The apparent discrepancies between these reports suggest that the relationship between NRF2 and p53 may well be dependent on the cellular and biological context.

### Targeting anti-oxidative stress proteins as possible anti-cancer therapy

Stabilization or reactivation of p53 throughout the use of small molecules is a promising therapeutic strategy [96, 98, 153-157]. However, we believe that alternative approaches should be explored in order to win the war against cancer [158-163].

In light of the model that NRF2 and p53 synergize in enhancing the cellular anti-oxidative stress mechanisms, we reason that cancer cells that exhibit high NRF2 levels and harbor WT p53 will be more dependent on these pathways to sustain chemo resistance. It is therefore tempting to speculate that targeting the anti-oxidative stress modules, that are promoted by NRF2 and p53, in combination with chemotherapeutic that will increase ROS, such as Doxorubicin [164], is a rational approach for treating such cases. Some of potential drugable targets would be the glutaminolysis pathway, the glutathione generating pathway, anti-oxidative stress proteins and NRF2. As discussed above, p53 and NRF2 promote glutaminolysis that supplies the glutamate and NADPH to generate glutathione to battle oxidative stress (Fig. 1). Targeting Kidney type Glutaminase (KGA), an essential enzyme in glutaminolysis, using an inhibitor such as BPTES, could inhibit glutaminolysis [165-167]. Indeed, this compound was shown to inhibit growth of MYC transformed P493 cells *in vivo* by increasing ROS and reducing glutathione levels in these cells [166]. Another strategy to reduce glutathione is by targeting γ-glutaminase, a rate limiting enzyme in the generation of glutathione, using BSO, a compound that has been shown to be well tolerated in man [168].

Piperlongumine has been shown to be selectively toxic to cancer cells and its mechanism of action was proposed to involve enhancing ROS in cancer cells by binding to a wide number of anti-oxidative stress proteins [169]. It is therefore plausible that piperlongumine could be useful in treating cancers that rely on anti-oxidative stress proteins that are induced by NRF2 and p53. Using 80 piperlongumine analogs it was recently demonstrated that the toxic effect of piperlongumine is due to its activity in crosslinking glutathione to proteins and depletion of cellular glutathione [170].

A more direct approach could be the targeting of NRF2 itself using brusatol, a natural compound that was shown to inhibit NRF2 by promoting its degradation [171]. The same study showed that brusatol synergized with chemotherapeutic agents *in vitro* and in xenograft models and to induce death in tumors that have acquired drug resistance through NRF2.

It is now clear that the cancer cells will utilize endogenous protective mechanisms to evolve chemoresistance [172-174]. It is therefore important that we take a close look at what we believe are tumor suppressor proteins and pathways as they might paradoxically be hijacked by cancer cells to promote their growth and survival. NRF2 and p53 may well be the two-faced Januses of cancer.
